# TDP43 interacts with MLH1 and MSH6 proteins in a DNA damage-inducible manner

**DOI:** 10.1186/s13041-024-01108-3

**Published:** 2024-06-05

**Authors:** Vincent E. Provasek, Manohar Kodavati, Brandon Kim, Joy Mitra, Muralidhar L. Hegde

**Affiliations:** 1 Division of DNA Repair Research within the Center for Neuroregeneration, Department of Neurosurgery, Houston Methodist Research Institute, Houston, TX 77030 USA; 2 School of Medicine, Texas A&M University, College Station, TX 77843 USA; 3 Department of Neuroscience, Weill Cornell Medical College, New York, NY 10065 USA; 4 Department of Neuroscience, Rice University, Houston, TX 77006 USA

**Keywords:** TDP-43, DNA mismatch repair (MMR), DNA double-strand breaks (DSBs), Neurodegeneration, Amyotrophic lateral sclerosis (ALS), Co-immunoprecipitation (CoIP), Proximity ligation assay (PLA)

## Abstract

Amyotrophic lateral sclerosis (ALS) is a fatal neurodegenerative disease that affects the motor neuron. One aspect of the neuropathology involved in ALS includes increased genomic damage and impaired DNA repair capability. The TAR-DNA binding protein 43 (TDP43) has been associated with both sporadic and familial forms of ALS, and is typically observed as cytosolic mislocalization of protein aggregates, termed TDP43 proteinopathy. TDP43 is a ubiquitous RNA/DNA binding protein with functional implications in a wide range of disease processes, including the repair of DNA double-strand breaks (DSBs). While TDP43 is widely known to regulate RNA metabolism, our lab has reported it also functions directly at the protein level to facilitate DNA repair. Here, we show that the TDP43 protein interacts with DNA mismatch repair (MMR) proteins MLH1 and MSH6 in a DNA damage-inducible manner. We utilized differentiated SH-SY5Y neuronal cultures to identify this inducible relationship using complementary approaches of proximity ligation assay (PLA) and co-immunoprecipitation (CoIP) assay. We observed that signals of TDP43 interaction with MLH1 and MSH6 increased significantly following a 2 h treatment of 10 μM methylmethanesulfonate (MMS), a DNA alkylating agent used to induce MMR repair. Likewise, we observed this effect was abolished in cell lines treated with siRNA directed against TDP43. Finally, we demonstrated these protein interactions were significantly increased in lumbar spinal cord samples of ALS-affected patients compared to age-matched controls. These results will inform our future studies to understand the mechanisms and consequences of this TDP43-MMR interaction in the context of ALS-affected neurons.

**Supplementary Information:**

The online version contains supplementary material available at 10.1186/s13041-024-01108-3.

## Main Text

Amyotrophic lateral sclerosis (ALS) is a severe neurodegenerative disease characterized by progressive loss of both upper and lower motor neurons of the central nervous system (CNS). Over 90% of cases are sporadic and exhibit a complex etiology. Approximately 10% of cases are due to familial mutations in various genes, including *Tardbp,* which encodes the TDP43 protein [1]. TDP43 is a nuclear protein consisting of multiple domains including a nuclear localization sequence, two RNA-recognition motifs, and one low complexity domain (LCD) within the C-terminus. TDP43 utilizes its RRM domains to bind both RNA and DNA, allowing it to exert its extensive role in regulating RNA metabolism. The LCD primarily mediates protein–protein interactions (PPIs) and is also the site of most familial ALS-linked mutations [2]. Moreover, the LCD contains an amyloidogenic region that is critical for the cytotoxic aggregation observed in ALS [3]. These characteristics are important for TDP43’s various cellular functions, but also make TDP43 a factor in several facets of ALS pathology. In fact, TDP43 has been implicated in over 95% of ALS cases where it presents cytosolic mislocalization as insoluble, cytotoxic aggregates; this phenomenon has been termed TDP43 proteinopathy [1].

The effects of TDP43 proteinopathy are typically categorized into two classes: loss of nuclear function and gain of cytosolic toxicity. Our lab has focused on the former and has recently shown that TDP43 is a requisite co-factor for neuronal DNA double-strand break (DSB) repair. Specifically, TDP43 is recruited to the DSB site with other DNA damage response (DDR) factors where it binds DNA and facilitates nonhomologous end joining (NHEJ) repair by acting as a scaffold for X-ray repair cross-complementing protein 4 (XRCC4) and DNA Ligase 4 (Lig4) [4].

Furthermore, our lab has recently reported the regulatory relationship between TDP43 and MMR factors at the RNA level (Preprint [5]). We showed that TDP43 regulates the expression of multiple MMR proteins with implications for DNA damage and repair. Whether TDP43 interacts with or otherwise affects MMR at the protein level remains unexplored. Other reports linking MMR proteins to DSB repair have placed renewed importance on understanding the PPIs between TDP43 and MMR [6]. Furthermore, it has been reported that both MLH1 and MSH6 play both direct and indirect roles in DSB repair [7, 8]. Given our previous work showing the role of TDP43 in regulating MMR expression, we questioned whether TDP43 may also affect MMR at the protein level, particularly in the context of DNA damage.

To investigate whether TDP43 interacts with MMR factors MLH1 and MSH6, we utilized two complementary approaches for identifying PPIs: proximity ligation assay (PLA) and co-immunoprecipitation assay (CoIP). These methods provide both sensitive and specific means of identifying PPIs in vitro, respectively. In each case, we utilized human neuroblastoma SH-SY5Y cells (ATCC) cultured in standard conditions using DMEM/F12 media (Gibco) supplemented with 10% Fetal Bovine Serum (Sigma) and 1% Penicillin–Streptomycin (Sigma). Cells subsequently underwent a 72 h differentiation protocol with media supplemented with retinoic acid (RA) (10 μM) and BDNF (50 ng/mL) prior to all experiments.

**Fig. 1:**
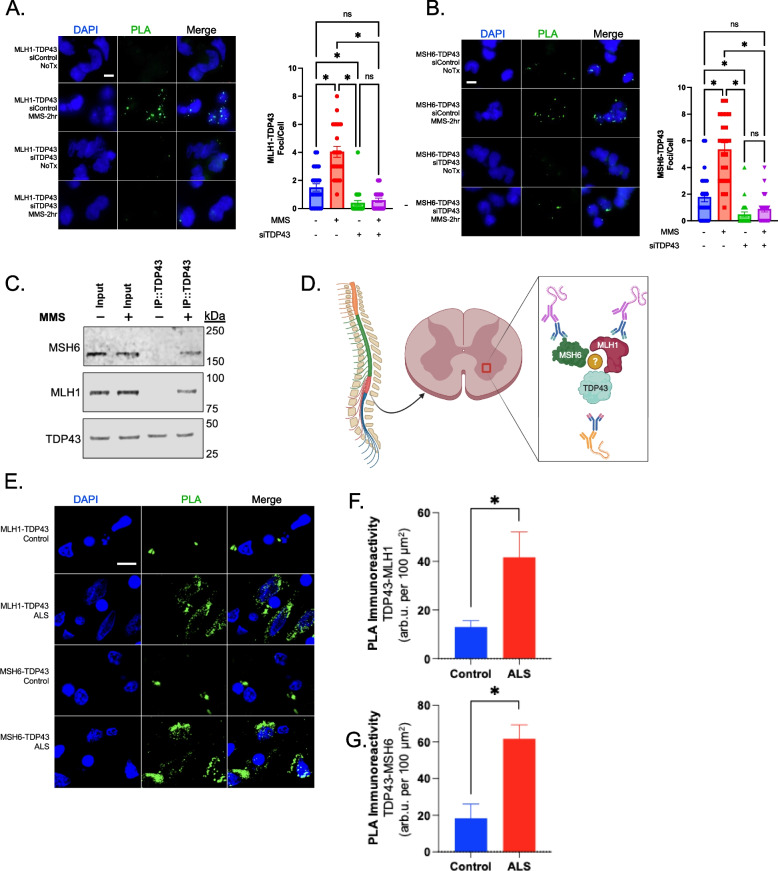
TDP43 Interacts with MMR factors in a DNA Damage-Inducible Manner. **A** Representative immunofluorescence (IF) images and quantitation of proximity ligation assay (PLA) in neuronal cell cultures show a significant increase in the number of foci per nucleus for TDP43-MLH1 interaction following methylmethanesulfonate (MMS) treatment. This interaction is abolished by TDP43 knockdown. **B** Representative IF images and quantitation of PLA in neuronal cell cultures show a significant increase in the number of foci per nucleus for TDP43-MSH6 interaction following MMS treatment which is also abolished by TDP43 knockdown. **C** Western blot analysis of co-immunoprecipitation assay. Pulldown of TDP43 and its interacting proteins show detection of both MLH1 and MSH6. **D** Schematic showing the ventral horn region of the human lumbar spinal cord used to measure TDP43-MMR protein interaction. **E** Representative IF images of PLA showing increased TDP43-MLH1 and TDP43-MSH6 interaction within the ventral horn of the lumbar spinal cord of ALS-affected tissue compared to age-matched controls. **F**, **G** Quantitation of PLA immunoreactivity. Scale bars, 5 µm. p

To identify the protein interactions between TDP43 and MLH1 or MSH6, we first utilized the Duolink PLA kit (Sigma) with cells seeded on 8-well chamber slides (Millicell EZ slides, Millipore). Cultures were grown for 48 h prior to treatment with 10 μM methylmethanesulfonate (MMS) (Sigma) for 2 h prior to fixation for 15 min at 37 °C using 4% paraformaldehyde. After permeabilization (0.5% Triton X-100), Primary antibodies (MLH1 ProteinTech Cat. 11,697–1-AP, MSH6 ProteinTech Cat. 18,120–1-AP, TDP43 ProteinTech Cat No. 10782–2-AP) and PLA plus/minus probes were incubated with cells for 1 h prior to ligation for 30 min at RT. Slides were washed and then amplification was performed for 100 min at 37 °C. Cells were counter-stained with DAPI and analyzed by fluorescence microscopy (Zeiss). Foci per cell were counted by three blinded assistants and the values averaged. The results of PLA experiments are shown in Fig. 1A, B. Cell cultures treated with control siRNA (Thermo, Cat #4390843) displayed minimal PLA signals of 1.7 and 1.9 foci/cell for TDP43-MLH1 and TDP43-MSH6 pairs, respectively. MMS-induced DNA damage significantly increased this interaction with an average of 4.1 and 5.7 foci/cell for the TDP43-MLH1 and TDP43-MSH6 pairs, respectively. The specificity of the PLA probes was confirmed by siRNA-mediated TDP43 knockdown (Thermo, Cat #4390771) for both MLH1 and MSH6 experiments. We also performed a positive control experiment testing for the DNA damage-inducible interaction between TDP43 and XRCC4 (Supplementary Figure 1A-B). Expectedly, MMS treatment significantly increased interaction between TDP43 and XRCC4, presumably at sites of secondary DSBs.

We next performed CoIP experiments to further investigate the interaction between TDP43 and MLH1 and MSH6 in neuronal cell cultures. We utilized the Pierce Co-Immunoprecipitation Kit (Thermo) performed according to the manufacturer’s instructions. Briefly, 1 mg of total lysate was incubated overnight at 4 °C with a resin column containing covalently linked anti-TDP43 antibody to immunoprecipitated TDP43 and its associated proteins from total cell lysates. We then analyzed the immunoprecipitates by Western blotting with anti-MLH1 and anti-MSH6 antibodies. As shown in Fig. 1C, we detected a specific band corresponding to MLH1 and MSH6 in the MMS-treated TDP43 immunoprecipitates, but not in the control immunoprecipitates. This indicates that TDP43 interacts with MLH1 and MSH6 in a DNA damage-inducible manner. To confirm the specificity of the interaction, we also performed a reverse CoIP experiment using anti-MLH1 and anti-MSH6 antibodies to immunoprecipitated TDP43 and its associated proteins. The presence of MLH1 and MSH6 were observed in TDP43 CoIP only from MMS-treated samples as shown in Supplementary Figure 1C. These results confirm that TDP43 interacts with MLH1 and MSH6 following DNA damage and emphasize the need to further explore the functional consequences of TDP43-MMR protein interactions on neuronal DNA repair.

We next questioned whether the DNA damage-inducible interaction we observed in vitro also occurred in the cortical tissue of ALS-affected patients (Fig. 1D). Patient characteristics are provided in Supplementary Table 1. TDP43 proteinopathy and DSB damage are both salient features of ALS-affected neurons, and our lab has also shown that TDP43 likely contributes directly to the formation of these DSBs [9]. We hypothesized that TDP43 would demonstrate increased interaction with MLH1 and MSH6 in diseased spinal cord tissues compared to controls. We used PLA to test this hypothesis in the same manner as our in vitro approach. We found that the PLA signal for TDP43-MLH1 and MSH6 interactions was significantly elevated in ALS tissues (Fig. 1E-G). Interestingly, we also observed low levels of interaction within the nuclei of aged control tissues, suggesting these protein interactions may also occur in aged CNS tissues.

Taken together, this study reports, for the first time, a DNA damage-inducible interaction between TDP43 and the MMR factors MLH1 and MSH6. Although not a comprehensive analysis of TDP43-MMR interaction, our study provides an important first step toward uncovering the newly appreciated relationship between TDP43 and the MMR pathway. Future studies will be required to characterize how TDP43 interacts with the selected MMR factors and confirm whether these interactions affect the DNA damage and repair landscape, especially in the neurodegenerative context.

### Supplementary Information

Additional file 1: Supplementary Fig. 1. A) Proximity ligation assay (PLA) immunofluorescence imaging of TDP43-XRCC4 in HEK293 cells show DNA damage induction significantly increased interaction. B) Histogram showing quantitation of TDP43-XRCC4 PLA foci per nucleus. C) Reverse co-immunoprecipitation immunoblotting shows interaction immunoprecipitation of MLH1 and MSH6 allows detection of TDP43 bands in MMS-treated cell cultures.

Additional file 2: Supplementary Table 1. Details of patient tissue specimens.

## Data Availability

Requests for materials and correspondence should be addressed to M.L.H. (mlhegde@houstonmethodist.org) or V.E.P. (vprovasek@houstonmethodist.org).
