# Supplementary figures and images for: TDP43 interacts with MLH1 and MSH6 proteins in a DNA damage-inducible manner
Source: Mol Brain. 2024 Jun 5;17:32. doi: 10.1186/s13041-024-01108-3 (PMC11155029; doi:10.1186/s13041-024-01108-3)

## Additional file: Supplementary Figure 1

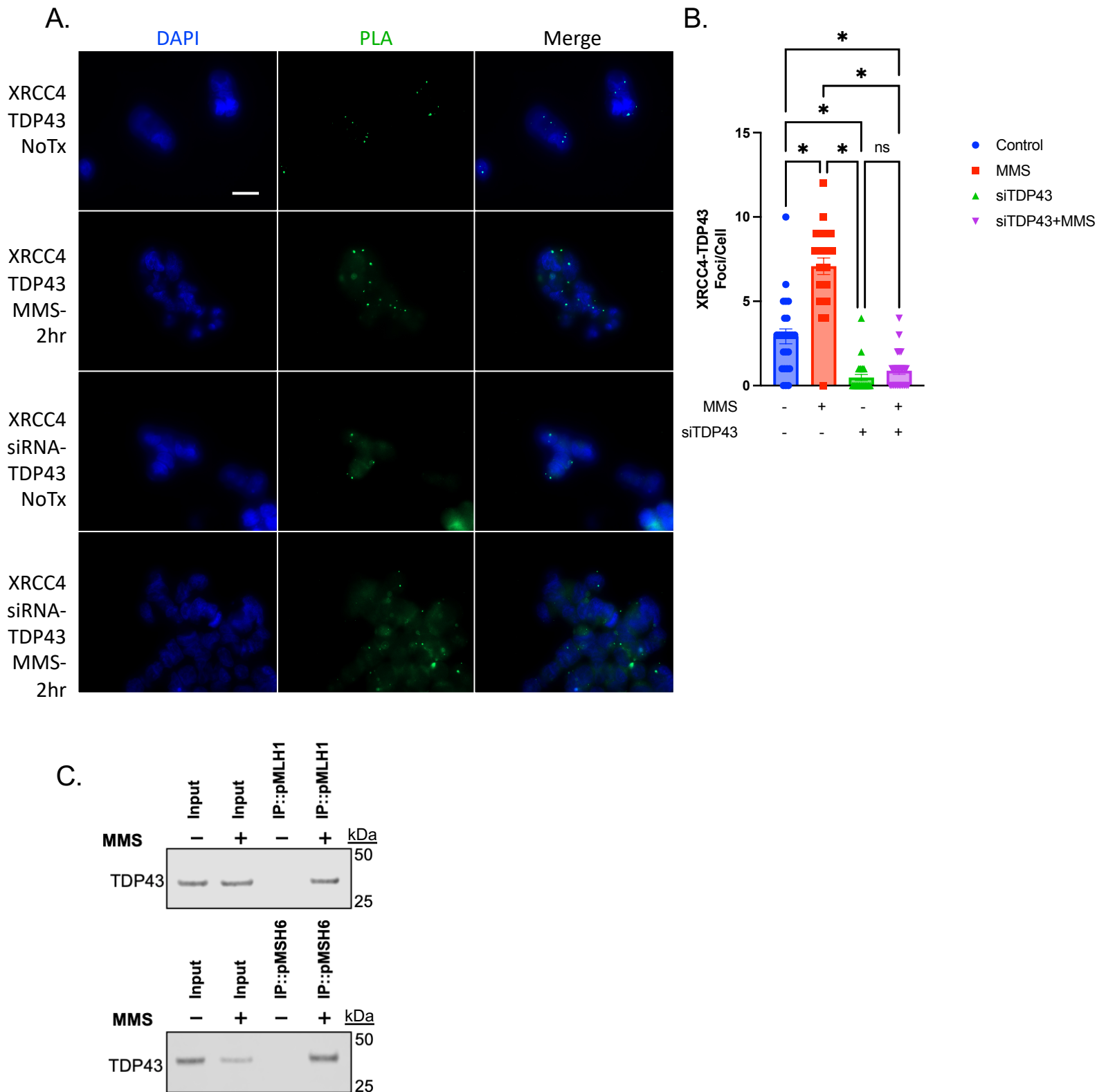

Supplement: Supplementary file 1 — Additional file 1: Supplementary Fig. 1. A) Proximity ligation assay (PLA) immunofluorescence imaging of TDP43-XRCC4 in HEK293 cells show DNA damage induction significantly increased interaction. B) Histogram showing quantitation of TDP43-XRCC4 PLA foci per nucleus. C) Reverse co-immunoprecipitation immunoblotting shows interaction immunoprecipitation of MLH1 and MSH6 allows detection of TDP43 bands in MMS-treated cell cultures. [file 13041_2024_1108_MOESM1_ESM.pdf]
