# Supplementary material for: TDP43 interacts with MLH1 and MSH6 proteins in a DNA damage-inducible manner
Source: Mol Brain. 2024 Jun 5;17:32. doi: 10.1186/s13041-024-01108-3 (PMC11155029; doi:10.1186/s13041-024-01108-3)
Supplement: Supplementary file 2 — Additional file 2: Supplementary Table 1. Details of patient tissue specimens. [file 13041_2024_1108_MOESM2_ESM.pdf]

Additional file: Supplementary Table 1. Details of patient and control tissue.

Category	Identification No.	Age	Sex	Ethnicity	PMI-cr (hrs)	PMI-f (hrs)
Control	110023	71	F	Caucasian	2.4	61.9
ALS	120018	58	F	Caucasian	1.0	41.5
